# Evaluation of Contact Heat Transfer Coefficient and Phase Transformation during Hot Stamping of a Hat-Type Part

**DOI:** 10.3390/ma8042030

**Published:** 2015-04-22

**Authors:** Heung-Kyu Kim, Seong Hyeon Lee, Hyunjoo Choi

**Affiliations:** 1 Department of Automotive Engineering, Kookmin University, 77, Jeongneung-ro, Seongbuk-gu, Seoul 136-702, Korea; 2 Graduate School of Automotive Engineering, Kookmin University, 77, Jeongneung-ro, Seongbuk-gu, Seoul 136-702, Korea; E-Mail: csjijon@naver.com; 3 School of Advanced Materials Engineering, Kookmin University, 77, Jeongneung-ro, Seongbuk-gu, Seoul 136-702, Korea; E-Mail: hyunjoo@kookmin.ac.kr

**Keywords:** phase transformation, workpiece, hot stamping

## Abstract

Using an inverse analysis technique, the heat transfer coefficient on the die-workpiece contact surface of a hot stamping process was evaluated as a power law function of contact pressure. This evaluation was to determine whether the heat transfer coefficient on the contact surface could be used for finite element analysis of the entire hot stamping process. By comparing results of the finite element analysis and experimental measurements of the phase transformation, an evaluation was performed to determine whether the obtained heat transfer coefficient function could provide reasonable finite element prediction for workpiece properties affected by the hot stamping process.

## 1. Introduction

Hot stamping is a key technology for providing high-strength lightweight steel car body parts that led to the development of fuel-efficient cars [1,2]. While satisfying the crashworthiness regulation as well as providing reduced springback, more complex and thinner parts can be produced by the hot stamping process [3,4]. The hot stamping process requires a high degree of process control technology to produce automotive components with complex shapes and high mechanical quality [5].

The quality of the final product is affected by various process conditions during the hot stamping thermo-mechanical cycle. Hence, the process technology for hot stamping has been advanced by using Finite Element simulation [6,7]. In particular, the thermal aspects, which are affected by heat transfer at the interface between the die and the workpiece, are of foremost importance since they determine the rheological behavior [3,8], the phase transformation [3], and the transformation plasticity [9] of the steel.

To date there have been some investigations regarding the interface heat transfer at the contact surface. For the forging processes, Nshama [10] evaluated the heat transfer conditions on the metal forming interface of a billet by comparing the results of measured temperatures from experiment to that from finite element simulation. Similarly, Lenhard *et al.* [11] estimated the workpiece-die heat transfer coefficient for a warm forging process by comparing the FEM simulation with the experimental measurement of die temperatures. In case of the hot stamping of sheet metals, Geiger *et al.* [12] determined the interface heat transfer coefficient as a linear function of contact pressure for a sheet metal workpiece placed between flat plates and examined its validity by applying it to the finite element analysis of a cup drawing test. The limitation of their investigation is that the nonlinear dependence of heat transfer coefficient on pressure was not examined. Bosetti *et al.* [13] identified the interface heat transfer coefficient dependence on the applied pressure for the pressure range between 0 and 40 MPa. They considered a simple testing procedure where metal blanks are compressed between flat dies. In the present investigation, however, we considered the industrial hot stamping process using a hat-type die, which can be used for manufacturing automotive body reinforcement parts such as side sill, and obtained the interface heat transfer coefficient based on the inverse analysis technique.

The hot stamping process is generally composed of the press forming stage, the die quenching stage, and the air cooling stage. For integrated finite element analysis of the hot stamping process, a heat transfer model usable for press forming as well as for die quenching needed to be established. For that purpose, we tried to model the heat transfer coefficient for a hot stamping process of a hat-type sheet metal product. First, an experimental hot stamping process was conducted and temperature was measured at selected locations on the hot stamping die. Workpiece properties including the martensite phase ratio were measured using optical microscopy, an X-ray diffractometer, and a Vickers hardness tester. The interface heat transfer coefficient was assumed as a function of pressure on the contact surface and estimated based on temperature data collected during the forming process by using a simplified inverse analysis. The heat transfer coefficient for the die quenching stage was also examined. The function obtained for the forming stage was applied as the interface heat transfer coefficient for finite element analysis and evaluated based on the martensite phase ratio measured by optical microscopy and X-ray diffraction. From the comparison, the performance of the present heat transfer coefficient models was evaluated.

## 2. Hot Stamping Experiment and Measuring of Workpiece Properties

### 2.1. Experimental Set-up and Hot Stamping Process 

**Figure 1:**
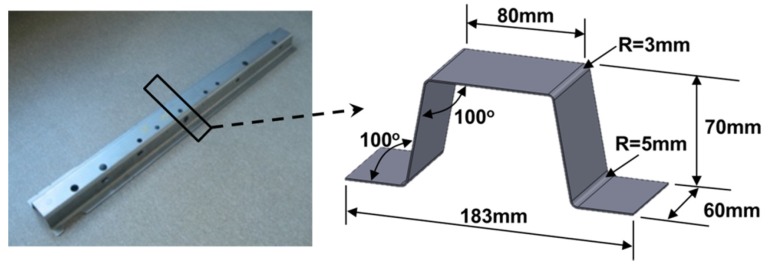
A hat-type product design for the present hot stamping test.

The target product of the present hot stamping investigation is the hat-type sheet metal product shown in Figure 1. This product was designed based on the sectional shape of a side sill, which is one of the high-strength safety parts of an automotive body structure. Due to the simplicity of the shape, two-dimensional finite element simulation can be used to analyze the forming process.

**Figure 2:**
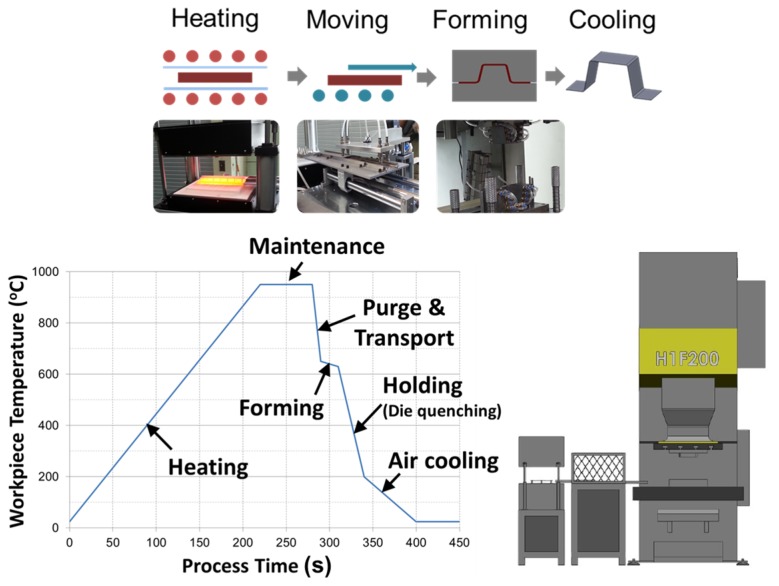
Schematic of hot stamping process and schematic time-temperature profile of the hot stamping process for the hat-type product.

A high-strength steel (SABC1470, the chemical composition is shown in Table 1) sheet with a thickness of 1.22 mm was hot-formed in a hat-type product forming die. Considering the thickness of sheet, the clearance between the side wall of the punch and die was set to be 1.22 mm at the lowest position of the die. In the present die setting, the upper die (punch) moved up and down and the lower die was stationary. The surface roughness of the workpiece was measured as R_a_ 6.3 μm. This forming technique consists of a couple of stages as described in Figure 2. First, the sheet metal workpiece was heated to the austenitizing temperature of 950 °C at a heating rate of 4.32 °C/s, and then held for 1 min to remove thermal gradients. Subsequently, the workpiece was purged and transferred from the furnace to the press, as fast as possible, to take advantage of the excellent formability at high temperatures. During purging and transport, the workpiece was cooled down to 650 °C. During forming, the workpiece was deformed at 650 °C at a forming speed of 54 mm/s and then was held for 5 s. To suppress the rising die temperature, cooling channels were installed in the die. Owing to the cooling effect by circulating water, the die temperatures maintained by the cooling channels were about 11 °C. A schematic of the process stages and the corresponding time-temperature profile are shown in Figure 2. The 200-ton hydraulic servo-press (model: KOMATSU H1F200) was used for hot stamping process.

**Table 1:** Chemical composition of the SABC1470 steel used in the hot stamping test.

Composition (wt.%)						
C	Si	Mn	P	S	B	Fe
0.23	0.26	1.24	0.015	0.002	0.0023	Balanced

### 2.2. Workpiece Properties by Hot Stamping

**Figure 3:**
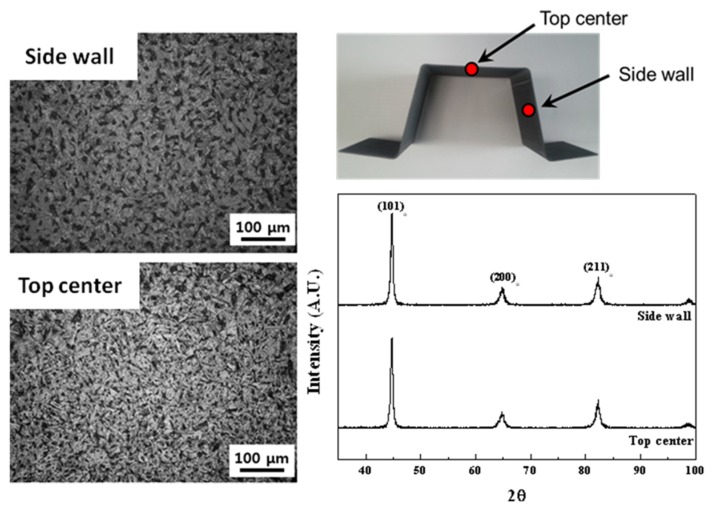
Optical micrograph picture and X-ray diffraction (XRD) data of the hat-type product formed by hot stamping.

To investigate microstructures with a low magnification of up to 200×, optical microscopy (OM) was used. The sheet metal workpiece was sectioned, mounted, and mechanically polished using SiC papers (up to # 2000) and a buffer. Afterward, the workpiece was etched with a 3% Nital solution. The austenite fraction was checked using X-ray diffraction (XRD, Rigaku, CN2301) with a CuKα radiation source (λ = 1.5405 Ǻ). XRD patterns were collected typically over 30°–100° in 2θ. Vickers hardness was measured on the workpiece using an indenter load of 1 kgf. Quasi-static uniaxial tensile tests were conducted using an Instron-type machine with a constant strain rate of 1 × 10^−4^ s^−1^. Table 2 summarizes the processing condition, the volume of martensite, and resulting hardness of the workpiece. Micro-Vickers hardness was measured on the side and top of the hat-type formed workpiece. The hardness in the side wall (the inclined face) of the workpiece is similar to that in the top center (the horizontal face in the middle) of the workpiece. Thus, the mechanical properties are found to vary insignificantly according to the location. Figure 3 shows optical micrographs of the workpiece. Martensite has lath morphology while its plates are not integral and are serrated at their edges. Although typical hot-stamped steels exhibit lath martensite with retained austenite, retained austenite is not detected by XRD in the present study. The workpiece is found to contain martensite together with other body-centered cubic (BCC) structured phases such as bainite or pearlite.

**Table 2:** The processing conditions and mechanical properties of the specimens.

Processing conditions or properties	Test 1	Test 2
Forming Speed (mm/s)	3	54
Holding Time (s)	5	5
Micro-Vickers at Side Wall (Hv)	418.8	533.0
Micro-Vickers at Top Center (Hv)	440.8	537.3
Martensite volume at Side Wall (%)	73	87
Martensite volume at Top Center (%)	78	89

## 3. Modeling and Evaluation of Heat Transfer Coefficient for the Hot Stamping Process

### 3.1. Heat Transfer Coefficient during the Forming Stage

The heat transfer coefficient during the forming stage can be estimated quantitatively based on the temperature data of the workpiece. However, it is very difficult to directly measure the temperature of workpiece inside the closed die. Hence, the heat transfer coefficient was calculated using an inverse analysis, based on the measured die temperature at several different locations. To measure the die temperature during the process, a K-type thermocouple of radius 0.65 mm was attached to the die by a tap screw. The thermocouple-die contact points were located at 10 mm from the die surface. The electric signals from the thermocouples were measured in real time and interpreted into temperature values on a DAQ (data acquisition) system. The temperature data was stored as a function of time from the beginning of the forming stage. The acquisition rate for temperature was 10 Hz.

As described in Table 2, we conducted the forming tests at the two different speeds of 3 and 54 mm/s. Since the forming speed of 54 mm/s (process duration of 1.3 s) was too fast for temperature measurement, we measured the temperature during the forming test of the speed 3 mm/s (process duration of 23 s).

**Figure 4:**
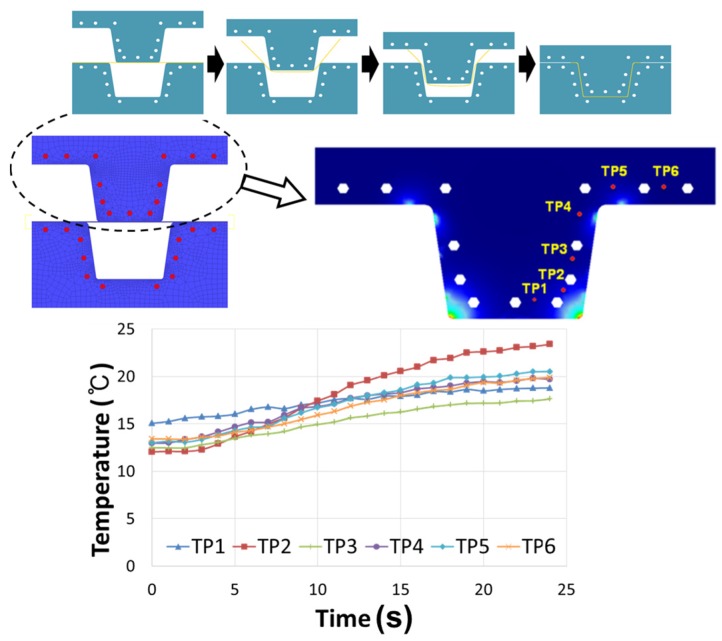
Temperature data measured at six points (TP1–TP6) on the upper die (punch) during forming at the speed of 3.0 mm/s.

We conducted the forming test repeatedly, and measured temperatures at TP1-TP6 during each test. The temperatures measured during a specific test are shown in Figure 4. The result shows that the initial temperatures at TP1 were rather higher than the other locations. It is because the die surface near TP1 contacted with the heated specimen for longer time than the other die surfaces during the previous tests.

Using finite element simulation, we searched for the heat transfer coefficient that predicted the temperature in best accord with the measurements. A commercial program (DEFORM-2D) was used for the finite element simulation. Based on the material database of DEFORM-2D, an elastic-plastic material model for SABC1470 was used. In addition, following the recommendation of DEFORM-2D for warm forming simulation, we used the shear friction of 0.25.

Compared with the inverse analysis for heat treatment problems without involving plastic deformation [14], formal inverse analysis considering the variant contact condition of press forming is very difficult. As an alternative to formal inverse analysis, we applied the DOE (design of experiment)-based inverse analysis approach to find the heat transfer coefficient based on the temperature measurement. By selecting appropriate ranges and levels for the design variables, we found an approximate optimum value for the heat transfer coefficient.

The error function to be minimized was defined as the sum of the squares of the temperature differences between the finite element prediction and the measurement at TP1–TP6. Then the error function was summed along the process time. As a result, the objective function is expressed by:

(1) $$Error=\sum_{t=t_{1}}^{t_{m}}{\left[\sum_{TP\_i=1}^{n}\left\{W_{TP\_i}\cdot {\left(T_{TP\_i}^{Exp}-T_{TP\_i}^{FEM}\right)}^{2}\right\}\right]}_{t}$$

where $T_{TP\_i}^{Exp}$ is the experimentally measured temperature at point TP_i and $T_{TP\_i}^{FEM}$ is the temperature predicted by finite element analysis at the same point. $W_{TP\_i}$ is the weighting factor for the temperature measurement data and was used to reflect the credibility or importance of each measurement. The mechanical contact between the die and the workpiece was mostly localized around the die corner regions during the forming stage. Therefore, temperatures measured near the die corners need to be considered more significantly than temperatures measured at other locations. Consequently, the weighting factors were assumed as $W_{1}$ = $W_{2}$ = $W_{3}$ = 1.0 and $W_{4}$ = $W_{5}$ = $W_{6}$ = 0.5.

The contact pressure between the die and the workpiece will influence the interface heat transfer coefficient on the contact surface. There have been some investigations about the dependence of the interface heat transfer coefficient on the contact pressure at the die-workpiece interface [10,11,12]. Inspired by these investigations, interface heat transfer coefficient $h_{c}$ was modeled as a power law function of contact pressure P as follows.

(2) $$h_{c}=a{\left(P/P_{0}\right)}^{b}+h_{0}\text{, Unit: }a\text{ [kW/}m^{2}\text{K], }P\text{ [MPa]}$$

where $P_{0}$ = 100 MPa is the reference contact pressure and the parameter a is the scaling parameter for the interface heat transfer coefficient. The parameter b determines the shape of the power law function. $h_{0}$ is the heat transfer coefficient for the condition of P = 0 and is assumed as the convection heat transfer coefficient of 0.02 kW/m^2^K [15,16]. This power law functional form can test various heat transfer coefficients although it cannot represent all kinds of detailed dependences. The value of contact pressure could be estimated from the normal stress predicted by finite element simulation. By assuming the die material as an elastic object, we could predict the normal stress based on the elastic deformation caused by contact with the sheet.

**Figure 5:**
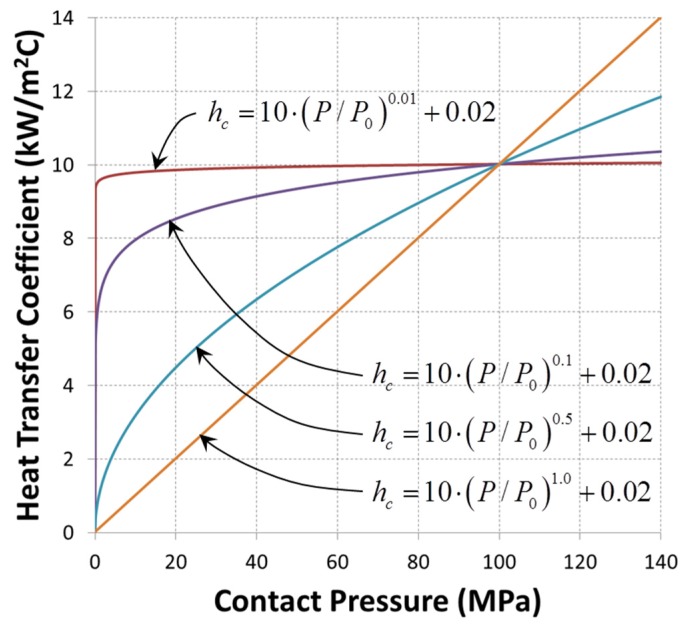
The shape of the power law function expressed by Equation (2).

There are two parameters, a and b, which should be estimated through inverse analysis. The tested ranges and levels for a and b are given in Table 3. For the case of a = 10, the shapes of the function $h_{c}$ are shown in Figure 5.

**Table 3:** Tested ranges and levels for *a* and *b*

Tested ranges	Tested levels							
8.0 ≤ *a* ≤ 11.0	8		9		10		11	
0.001 ≤ *b* ≤ 1.0	0.001	0.01		0.04	0.1	0.5		1

**Figure 6:**
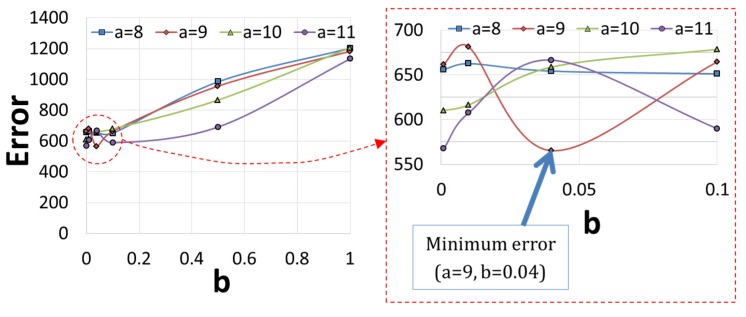
The variations of the objective function with respect to *a* and *b*

The objective function in Equation (1) was calculated based on the finite element simulation results obtained by various combinations of a and *b*. The variations of the objective function with respect to *a* and *b*. The variations of the objective function with respect to a and b are shown in Figure 6. The result shows that the objective function is minimum when using a = 9 and b = 0.04.

**Figure 7:**
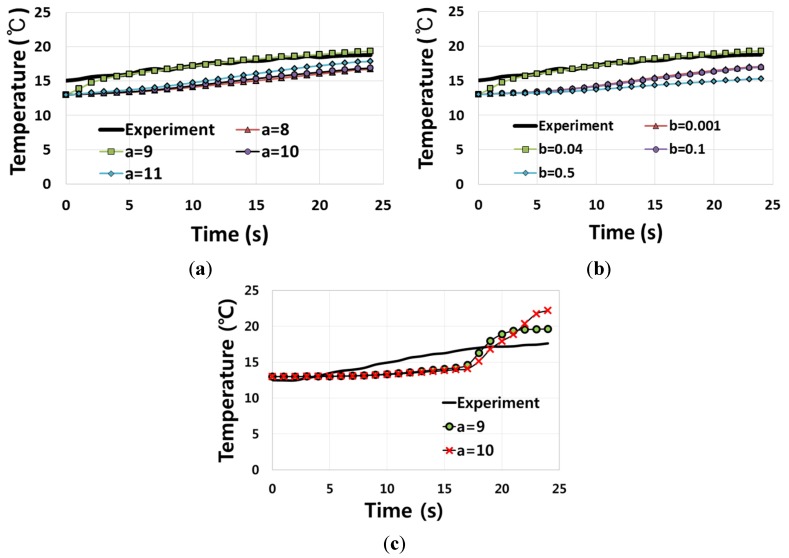
Temperature comparison between finite element predictions and experimental measurement (**a**) at TP1 when *a* varies with *b* = 0.04 fixed, (**b**) at TP1 when *b* varies with *a* = 9.0 fixed, and (**c**) at TP3 when (*a*, *b*) = (9.0, 0.04) and (*a*, *b*) = (10.0, 0.04).

In Figure 7, the finite element prediction of temperature for a = 9 and b = 0.04 at point TP1 is compared with those for the other combinations of a and b as well as for the experimentally measured temperatures. The result confirms that the temperature prediction for a = 9 and b = 0.04 is in better accord with the measured temperature than others.

**Figure 8:**
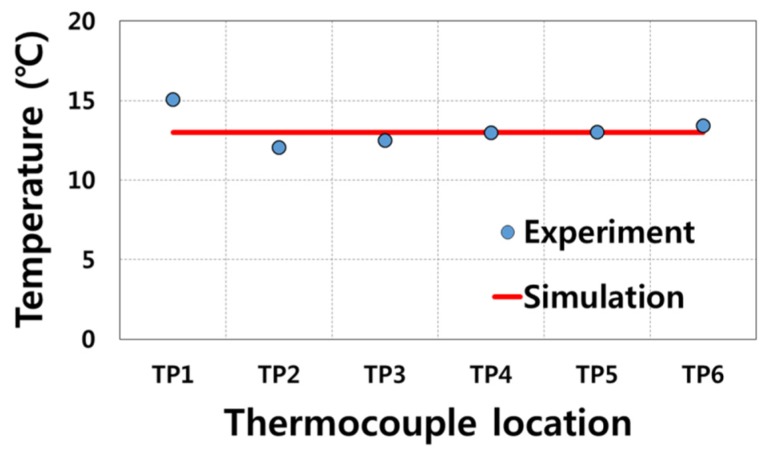
Comparison of the initial temperature condition for finite element simulation against the experimentally measured temperatures at TP1–TP6.

As already shown in Figure 4, the measured temperatures of die were slightly different depending on the thermocouple locations for TP1–TP6. The temperatures at the other locations of die can be assumed based on the temperatures at the thermocouple locations. To obtain accurate result from inverse analysis, the initial temperature distribution of die should be mapped into temperature of the element of the finite element model. However, identifying the initial temperature of die at all locations by using the temperatures at thermocouple locations is very difficult. Therefore, for the sake of computational convenience, we assumed that the initial temperature of all elements is 13 °C uniformly. The assumed initial temperatures for finite element simulation are compared with the measured temperatures at TP1–TP6 in Figure 8.

In the case where the punch speed is 54 mm/s, the duration of the forming stage is 1.3 s. The phase transformation during the forming stage is predicted by finite element simulation based on the interface heat transfer coefficient that uses *a* = 9 and *b* = 0.04. In the case of diffusion-controlled phase transformation, the volume fraction of transformed phase *i* from austenite is calculated based on the following Avrami type equation [17].

(3) $$X_{i}=X_{i}^{eq}\left(1-\exp\left(-B_{i}t^{n_{i}}\right)\right)$$

where $X_{i}^{eq}$ is the thermo-dynamical equilibrium fraction of phase *i*, and $B_{i}$ and $n_{i}$ are material constants to define transformation for phase *i*.

By using the Microstructure Module of DEFORM-2D, the hot stamping process can be simulated based on the phase transformation rule described above (http://www.deform.com). The material data for SABC1470 were obtained using JMatPro (http://www.sentesoftware.co.uk) and used as an input into the simulation using the Microstructure Module. In terms of CCT diagram of SABC1470, the martensite start temperature (M_s_) lies at 410 °C and the martensite finish temperature (M_f_) lies at 230 °C.

**Figure 9:**
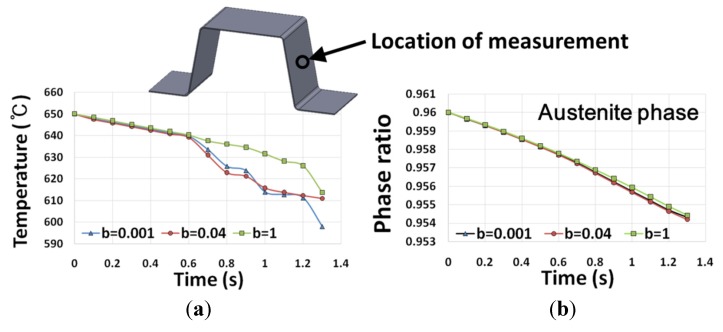
The evolutions of (**a**) temperature and (**b**) austenite phase ratio at a central location on the side wall.

Then the temperature as well as the phase transformation at the side wall was measured. The evolutions of temperature and austenite phase ratio at a central location on the side wall were plotted as a function of time in Figure 9. During the forming stage, the initial temperature of the workpiece of 650 °C dropped to 610 °C. The residual austenite phase ratio was 0.96 at the beginning of forming, which could be assumed based on the heat transfer analysis of the purge and transport process (duration of 10 s). After the forming stage, the residual austenite phase ratio decreased slightly to 0.954. The forming stage actually had little effect on the austenite phase ratio due to the very short process time. Although accurate modeling of the interface heat transfer coefficient is necessary for predicting the workpiece property after the forming stage, the effect of the heat transfer coefficient was very small in the case of the present forming process.

### 3.2. Heat Transfer Coefficient during the Holding (Die Quenching) and Air Cooling Stages

After the forming stage, the workpiece was held between the upper and lower dies to provide die quenching. Then the workpiece was moved outside the die for air cooling. The temperature as well as the phase transformation during the holding and air cooling stages was predicted by finite element analysis. A convection heat transfer coefficient of 0.02 kW/m^2^K was assumed for the air cooling stage.

The interface heat transfer coefficient of the power law function obtained for the forming stage was applied for finite element simulation of the holding stage. The finite element simulation result showed that the final martensite phase ratio after the holding and air cooling stages approached 84%. Compared with the experimentally measured phase ratio of 87%, the present finite element simulation provided a good prediction for the final martensite phase ratio. However, the increase rate of the martensite phase ratio might be too slow. Most of the martensite phase was predicted to be generated during the air cooling stage rather than the holding stage of 5 s.

**Figure 10:**
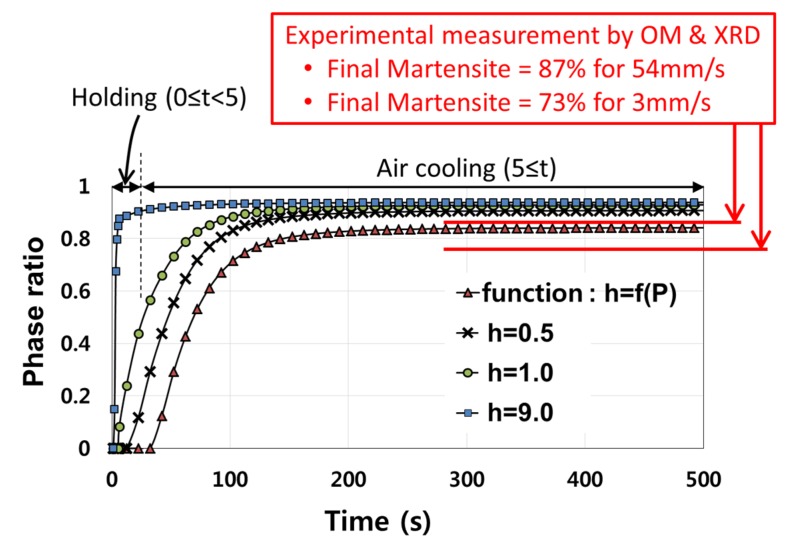
Martensite phase ratio predicted by finite element simulation based on various interface heat transfer coefficient models during holding and air cooling.

For comparison with the power law function, some constant values were applied as the interface heat transfer coefficient for the finite element simulation. Results showed that the interface heat transfer coefficient of constant 9 kW/m^2^K increased the final martensite phase ratio to 94% after holding and air cooling. Compared with the power law function, the increase rate of the martensite phase ratio was very high. Some other constant values were applied as the interface heat transfer coefficient. In the case of constant 0.5 kW/m^2^K, the final martensite phase ratio was predicted as 90%. The increase rate of martensite phase ratio was higher than the result obtained by the power law function. The evolution of the martensite phase ratio predicted by finite element simulation based on the various interface heat transfer coefficients is shown in Figure 10 compared with the measurements by OM and XRD.

**Figure 11:**
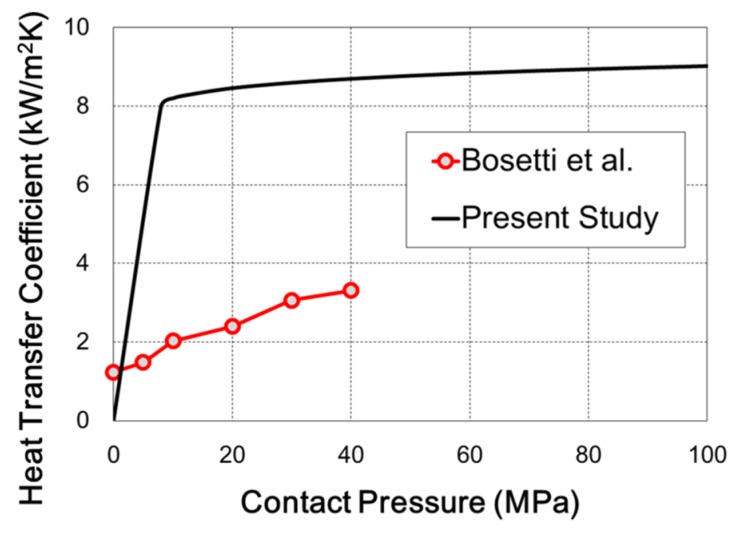
The comparison of heat transfer coefficient between the present study and Bosetti *et al.* [13].

The functional form of heat transfer coefficient (*a* = 9, *b* = 0.04) predicted in the present study is compared with the result from Bosetti *et al.* [13] in Figure 11. In the case of contact pressure 40 MPa, the heat transfer coefficient predicted in the present study is about three times that from Bosetti *et al.* [13] where heat transfer between a metal blank and two flat dies was estimated. However, the hat-type dies were used in the present hot stamping. In this case, the modeling of contact in the finite element analysis is not easy since the die-metal contact changes continuously and is not uniform. The heat transfer between die and blank depends on the element-to-element contact condition during finite element analysis. Therefore the heat transfer coefficient predicted in the present study is applicable to the finite element simulation of hot stamping processes involving the hat-type forming.

When the workpiece is deformed by punch action during the press forming stage, a significant contact pressure occurs on the contact surfaces. In the experiment of the present study, a displacement controlled servo-press was used to control the die movement. As a result, the contact pressure would decrease significantly at initiation of the holding stage because the dies are stationary during the holding stage and no further plastic deformation is required. Of course, the contact pressure would remain high during holding stage if a force controlled press machine is used. Owing to the decrease in contact pressure, the heat transfer coefficient modeled as a function of contact pressure seems to be very small. Accordingly, the contact pressure would increase when a sufficient volume change is caused by the martensite phase transformation. On the other hand, the interface heat transfer effect seems to be quite large when using the constant interface heat transfer coefficients. This is because a constant heat transfer coefficient would act even if contact pressure was very small. The present heat transfer coefficient model needs to be improved further to reflect the contact condition during the holding stage. For example, the parameter $h_{0}$ in Equation (2) might be used to represent the initial heat transfer coefficient corresponding to P = 0.

## 4. Conclusions

A heat transfer coefficient model was examined for the hot stamping process of a hat-type product. For the forming stage, the interface heat transfer coefficient on the contact surface was modeled as a power law function of contact pressure. The parameters of the power law function could be obtained based on the temperatures measured during a forming test by using inverse analysis. For the holding stage, the power law function as well as constants was used as the interface heat transfer coefficient in finite element simulations of the phase transformation. For this process, finite element simulation included an air cooling stage, which started after the die quenching stage. In the present investigation, the heat transfer coefficient was predicted to increase up to 8 kW/m^2^K at the contact pressure of 10 MPa. When using the predicted heat transfer coefficient value, the finite element simulation result of hot stamping showed that the final martensite phase ratio approached 84%. Comparison of the martensite phase ratio from finite element simulation (84%) against the OM/XRD-based measurement (73%) showed that the heat transfer coefficient obtained in the present invesitgation is a reasonable value. Of course, the present interface heat transfer coefficient model needs to reflect the characteristics during the holding stage.
